# Gamma Knife Irradiation of Injured Sciatic Nerve Induces Histological and Behavioral Improvement in the Rat Neuropathic Pain Model

**DOI:** 10.1371/journal.pone.0061010

**Published:** 2013-04-12

**Authors:** Yuki Yagasaki, Motohiro Hayashi, Noriko Tamura, Yoriko Kawakami

**Affiliations:** 1 Department of Physiology, School of Medicine, Tokyo Women’s Medical University, Tokyo, Japan; 2 Department of Neurosurgery, Neurological Institute, Tokyo Women’s Medical University, Tokyo, Japan; Friedrich-Alexander University Erlangen, Germany

## Abstract

We examined the effects of gamma knife (GK) irradiation on injured nerves using a rat partial sciatic nerve ligation (PSL) model. GK irradiation was performed at one week after ligation and nerve preparations were made three weeks after ligation. GK irradiation is known to induce immune responses such as glial cell activation in the central nervous system. Thus, we determined the effects of GK irradiation on macrophages using immunoblot and histochemical analyses. Expression of Iba-1 protein, a macrophage marker, was further increased in GK-treated injured nerves as compared with non-irradiated injured nerves. Immunohistochemical study of Iba-1 in GK-irradiated injured sciatic nerves demonstrated Iba-1 positive macrophage accumulation to be enhanced in areas distal to the ligation point. In the same area, myelin debris was also more efficiently removed by GK-irradiation. Myelin debris clearance by macrophages is thought to contribute to a permissive environment for axon growth. In the immunoblot study, GK irradiation significantly increased expressions of βIII-tubulin protein and myelin protein zero, which are markers of axon regeneration and re-myelination, respectively. Toluidine blue staining revealed the re-myelinated fiber diameter to be larger at proximal sites and that the re-myelinated fiber number was increased at distal sites in GK-irradiated injured nerves as compared with non-irradiated injured nerves. These results suggest that GK irradiation of injured nerves facilitates regeneration and re-myelination. In a behavior study, early alleviation of allodynia was observed with GK irradiation in PSL rats. When GK-induced alleviation of allodynia was initially detected, the expression of glial cell line-derived neurotrophic factor (GDNF), a potent analgesic factor, was significantly increased by GK irradiation. These results suggested that GK irradiation alleviates allodynia via increased GDNF. This study provides novel evidence that GK irradiation of injured peripheral nerves may have beneficial effects.

## Introduction

The gamma knife (GK) is well known as a minimally invasive surgical procedure that can control tumors without craniotomy [1], [2], [3], [4]. The mechanism of tumor control is thought to be a destructive effect. Recently, GK has also been performed for other disorders, such as trigeminal neuralgia, cancer pain and epilepsy, and its overall efficacy has been demonstrated in several clinical studies [5], [6], [7], [8]. In cases with these diseases, a destructive effect alone cannot explain the clinical efficacy obtained by GK irradiation. The GK-induced therapeutic effect was observed sooner than the predicted GK-irradiation-induced destructive effect [5], [9], [10]. Neuromodulatory effects induced by GK have been regarded as one of the factors curing, or at least reducing the severity of, the aforementioned diseases [5], [10], [11]. However, the mechanisms underlying GK-induced biological effects remain largely unknown. The histological effects of radiation in the normal brain and nerve tissues have been studied and radiation was shown to dose- and time-dependently induce focal neurodegenerative effects including necrosis [12], [13], [14]. Similarly, various alterations such as proliferation and morphological changes in astrocytes [15], [16], microglial activation [17], endothelial hyperplasia [16], and destruction of the blood brain barrier [12], [18] have also been reported without neurodegenerative effects or preceding necrosis. GK may exert neuromodulatory effects by inducing changes in non-neuronal cells.

Although morbid change has already occurred in tissues affected by the aforementioned diseases, the effects of GK on damaged neuronal tissue are still poorly understood. In several animal model studies, x-irradiation achieved remarkable recovery from spinal cord injury [19], [20], [21]. The mechanism of radiation effects is thought to be related to the production of reactive oxygen species [21]. However, the actual targets are still unknown. As a first step, a simple animal model which can be evaluated in a systematic manner is needed to study the effects of GK irradiation on injured nerves. We previously reported targeting protocols for the rat striatum using the magnetic resonance imaging (MRI) coordinate system with the Régis Valliccioni frame [22], [23]. Herein, we applied this protocol to rats with partial sciatic nerve ligation (PSL), a well-established neuropathic pain model [24], to examine the effects of GK on injured peripheral nerves. We examined whether application of GK to damaged peripheral nerves leads to histological and/or molecular changes, focusing especially on macrophage activation and restorative effects on the nerves. Then, we also determined changes in the pain responses of these model rats. This study was designed to examine the biological effects of GK irradiation on injured peripheral nerves using PSL model rats.

## Methods

### Animals

Adult male Wistar rats (Sankyo Lab Co., Tokyo, Japan) weighing 240–290 g were used. The animals were housed two per cage in a temperature controlled room under artificial illumination (9∶00–21∶00 h, lights on). Food and water were supplied *ad libitum*. Animal experiments conformed to the guidelines issued by the National Institutes of Health for Laboratory Animals. The present study was performed with approval from the Animal Experiments Committee of Tokyo Women’s Medical University (Certification No: 023).

### Neuropathic Pain Model

The rats were anesthetized with an intraperitoneal (i.p.) injection of pentobarbital (50 mg/kg). As first described by Seltzer et al, we created PSL models by tying approximately half of the right sciatic nerve with an 8-0 silk suture [24]. Muscle and skin layers were closed with a 4-0 silk suture, and the animals were allowed to recover from anesthesia. In sham-operated rats, the sciatic nerve was exposed without ligation.

### GK Irradiation

To apply GK irradiation to the rat sciatic nerve, we modified established GK irradiation methods for small animals, as reported previously [22], [23]. GK was performed one week after ligation or sham operation. Rats were anesthetized with pentobarbital (50 mg/kg, administered i.p.) and fixed in a Régis-Valliccioni frame (Neurospace, Neuilly, France), which allows determination of target points directly on MRI (Excelart 1.5-tesla unit, model MRT2001/P3; Toshiba Medical Systems Co., Tochigi, Japan) (Fig. S1A). Coronal steady-state free precession three-dimensional images were acquired using a 1.0 mm slice thickness with no gap and the matrix was 224 × 256. The repetition time, echo time and number of acquisitions were 12 msec, 6 msec and two, respectively. The images were transmitted to the Leksell Gamma Plan treatment planning system (Elekta Instrument AB, Stockholm, Sweden), where the three-dimensional coordinates of the target area were calculated and defined in the sciatic nerve region. A central maximum irradiation dose of 90 Gy was delivered to the right sciatic nerve using a Leksell GK model C unit (Elekta Instrument AB, Stockholm, Sweden) by means of a 4-mm collimator. We consistently used a 90 Gy dose, because a maximum central dose of 80∼90 Gy is used for clinical treatment of trigeminal neuralgia patients [5], [7]. The center of the irradiated area was calculated with reference to the structures in the thigh visible on MRI in all cases (Fig. S1B). In preliminary experiments, GK irradiation accuracy for the rat right sciatic nerve was confirmed by high-dose (maximum dose of 200 Gy) GK irradiation (Fig. S2).

### Immunoblotting

In total, 16 rats were used for determination of the effects of GK irradiation by immunoblotting. The rats were divided into four groups: sham, sham+GK, ligation and ligation+GK. GK was performed one week after ligation or sham operation and samples were collected three weeks after ligation. In total, 20 rats were used for time course analysis after ligation. The samples were obtained before ligation and at several time points thereafter (until seven weeks after ligation). The bilateral sciatic nerves were then quickly removed, under deep anesthesia with a high dose of pentobarbital, and frozen in liquid nitrogen. Frozen samples were homogenized in lysis buffer containing 137 mM NaCl, 20 mM Tris-HCl (pH 7.5), 1% NP40 and a protease inhibitor cocktail (Roche, Penzberg, Germany). The protein concentration was quantified using a BCA Protein Assay Kit (Thermo, IL, USA), and the same amount of total protein was assayed for each immunoblot. Primary antibodies were used at the following dilutions: anti-Iba-1, as a marker of macrophages and microglia [25], [26] (1∶1000, Wako, Osaka, Japan), anti-βIII-tubulin, as a marker of growing axons [27], [28], [29] (1∶5000, Millipore, MA, USA), anti-P0, as a marker of myelin in peripheral nerves [30] (1∶2500, Abcam, MA, USA), and anti-β-actin, as a loading control (1∶10000, SIGMA, MO, USA). The intensity of immunoreactivity was quantified using Java's freely available ImageJ software (NIH, MA, USA, http://rsb.info.nih.gov/ij). Data are presented as means ± SEM. The statistical significance of differences was determined using the unpaired t test and repeated measures ANOVA with Dunnett’s multiple comparisons test.

### Toluidine Blue Staining

In total, 35 rats were used for toluidine blue staining. The rats were divided into each group: intact, sham (1 w, 3 w, 7 w), sham+GK, ligation (1 w, 3 w, 7 w) and ligation+GK. GK was performed one week after ligation and samples were collected three weeks after ligation. After perfusion, the sciatic nerve was removed and post-fixed in 2% glutaraldehyde and 2% paraformaldehyde in 0.1% cacodylate buffer (pH 7.4) overnight. These sciatic nerve samples were then dissected and washed with cacodylate buffer three times, and approximately 10 mm of sciatic nerve including 3 mm proximal and 5 mm distal to the lesion site were collected. Nerves were cut into 2-mm long segments and post-fixed in 1% osmium tetroxide (OsO_4_) for 2 hr. After several washes with distilled water, the nerve segments were dehydrated through graded ethanol solutions (5 min each in 30%, 50%, 60%, 70%, 80%, 90%, 95%, twice in 95.5%, and twice in 100%). Samples were infiltrated in 100% ethanol and QY1 (NISSIN EM Co. Ltd., Tokyo, Japan) 1∶1, and the QY1 was replaced three times (5 min each). After overnight infiltration in QY1 and Epon812 resin 1∶1, the samples were polymerized in 100% Epon812 resin at room temperature for 6 hr. Samples were then embedded employing a silicon embedding plate and polymerized at 60°C for 3 days. Semi-thin cross sections (0.5 µm) of sciatic nerves 1 mm proximal (b, c, and d) to the injury site and 1 mm distal (e, f, and g) to injury site were stained with 0.5% toluidine blue. Pictures were taken from the center of the injured site with a LINCE virtual slide system (CLARO, Hirosaki, Japan) or BX-50 microscope (Olympus, Tokyo, Japan). The number and diameters of the myelinated fibers in the section were measured using Java's freely available ImageJ software. Data are presented as means ± SEM. The statistical significance of differences was determined using the unpaired t test and repeated measures ANOVA with Tukey’s multiple comparisons test and the Kolmogorov-Smirnov test.

### Immunohistochemistry

In total, 20 rats were used for immunohistochemistry. The rats were divided into four groups: sham, sham+GK, ligation and ligation+GK. GK was performed one week after ligation and samples were collected three weeks after ligation. The rats were deeply anesthetized with pentobarbital (50 mg/kg i.p.) and intracardially perfusion-fixed with freshly prepared 4% paraformaldehyde in 0.1 M phosphate buffer. After perfusion, the sciatic nerve was removed and post-fixed in 4% paraformaldehyde overnight, and then permeated with 25% sucrose in phosphate buffered saline (PBS) for one day and 40% sucrose in PBS for two days. These samples were then frozen in O.C.T. compound (Sakura Finetechnical, Tokyo, Japan) and stored at −80°C until use. Frozen samples were sectioned longitudinally with a cryostat at a 25 µm thickness. The sections were incubated with rabbit anti-Iba-1 polyclonal antibody (1/1000, Wako, Osaka, Japan) overnight at 4°C. Primary antibody was subsequently detected with a Vectastain Elite ABC-kit (Vector, CA, USA).

### Oil Red O Staining

To confirm the clearance of myelin debris, longitudinal sections of the sciatic nerve were stained with Oil Red O, a highly specific stain for degenerating myelin [31]. Sciatic nerve sections were prepared as described above. Sections were dehydrated and incubated in a 0.3% Oil Red O staining solution (Muto, Tokyo, Japan) for 15 min, rinsed in 60% isopropyl alcohol, washed in distilled water, and covered with an aqueous mounting medium.

### Quantification of Iba-1 Positive, Oil Red O Positive Areas, and Number of Iba-1 Positive Cells

An approximately 1.5 cm length of sciatic nerve, including proximal, injured (intact area in sham cases), distal1, and distal2 (each area about 2 mm) portions, was dissected from the rat and longitudinal sections (25 µm thickness) were created as described above. One of every 10 sections was selected (total 5∼8 sections/rat) for Iba-1 and Oil Red O staining. Four 250 µm squares were randomly selected from each portion of the (proximal, injured, distal1, and distal2) for examination of a section. The size of the Iba-1 or Oil Red O positive area was measured using Java’s freely available ImageJ software. The number of Iba-1 positive cells was determined using a cell counter with ImageJ software. Data are presented as means ± SEM. The statistical significance of differences was determined using the unpaired t test.

### Behavioral Testing: Mechanical Threshold in Rats

In total, 16 rats were used for behavioral testing. Rats were divided into four groups, sham, sham+GK, ligation and ligation+GK. GK was performed one week after ligation or sham operation. Withdrawal thresholds for mechanical stimulation of the rat hindpaw were measured using a ‘dynamic plantar aesthesiometer’ (Ugo Basile, Lombardia, Italy), which is an automated Von Frey type system. The mechanical stimulation, which is applied with a plastic filament (0.5 mm diameter), gradually increases in force from 0 g to the withdrawal threshold level. The rate of the force increase was 2.5 g/s. The maximum force was 25 g. Each paw was measured alternately after more than 5 min. The threshold of paw withdrawal in response to a mechanical stimulus was determined as the average of three measurements per paw. The measurements were performed before ligation and at several time points thereafter (until 9 weeks after ligation). Data are presented as means ± SEM. The statistical significance of differences was determined by the paired t test and time-dependent ANOVA with Tukey’s post hoc test.

## Results

### Morphological Changes in the Sciatic Nerve after PSL

**Figure 1:**
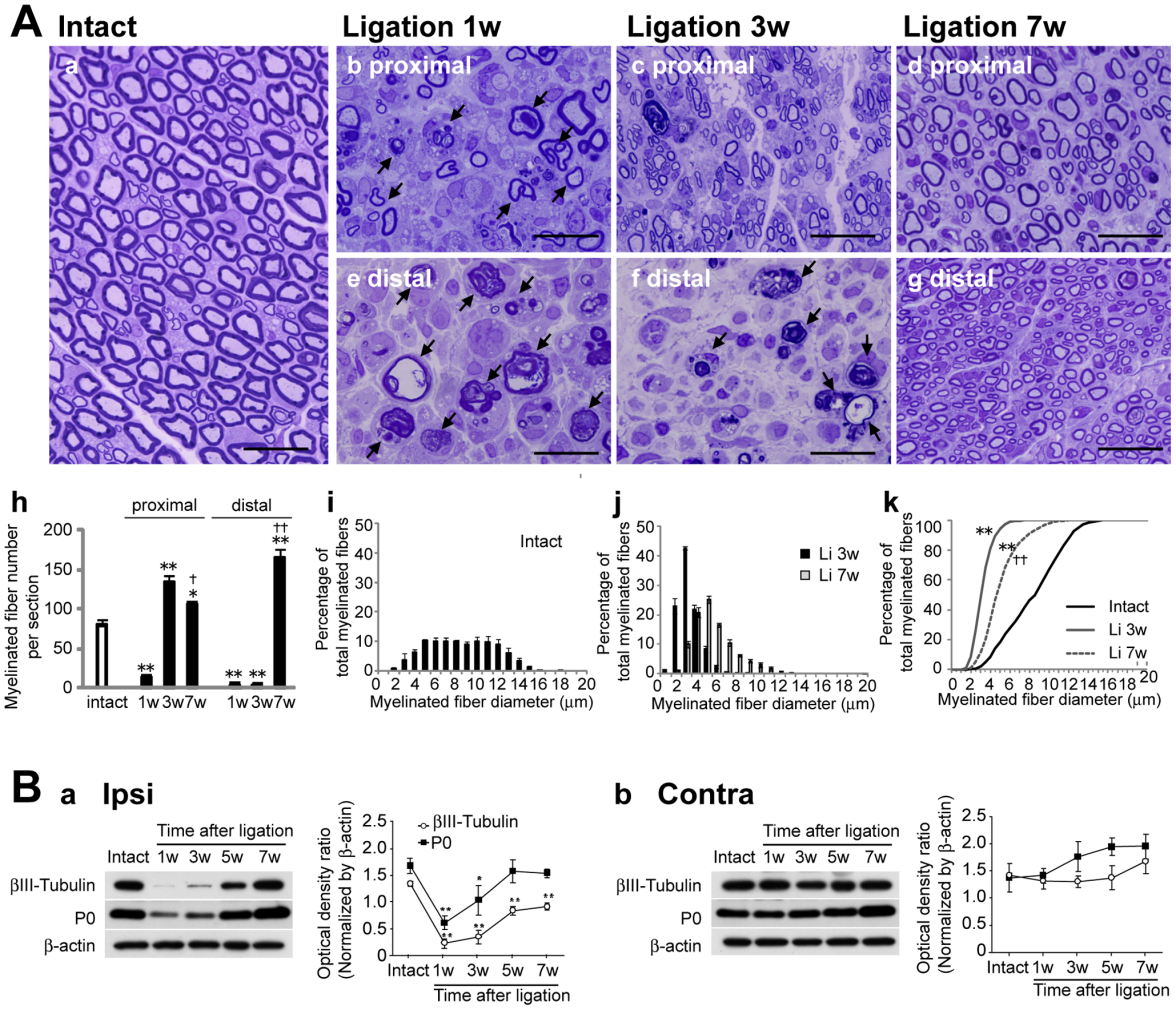
Toluidine blue staining of injured nerves following partial sciatic nerve injury. **A,** Time course analysis of morphological changes in the injured nerve. **a–g**; Semi-thin cross sections (0.5 µm) of sciatic nerves 1 mm proximal (b, c, and d) to the injury site and 1 mm distal (e, f, and g) to the injury site were stained with 0.5% toluidine blue. Representative photographs of cross sections of sciatic nerves. **a;** intact, **b and e;** one week after ligation, **c and f;** three weeks after ligation, **d and g;** seven weeks after ligation. Scale bars; 20µm for each panel. Arrow; myelin vacuolation and degenerating myelin. **h,** Density of myelinated fibers per section from each rat. **p<0.01, *p<0.05 compared with intact group. ††p<0.01, †p<0.05 compared with 3 weeks after ligation group. **i and j,** Distribution of myelinated fiber diameters is shown as a histogram with 1µm bin. **i;** Intact, **j;** Injured nerve. Data are presented as means ± SEM. **k,** Plot of the cumulative myelinated fiber diameter distribution. Black line; intact rat (*n* = 2264 fibers), gray line; 3 weeks after ligation (*n* = 6179 fibers), gray broken line; 7 weeks after ligation (*n* = 2510 fibers). **p<0.01 compared with intact group. ††p<0.01 compared with 3 weeks after ligation group. **B,** Immunoblot analysis of βIII-tubulin and P0 protein expressions after ligation. The top blots are examples of immunoreactive bands against anti-βIII-tubulin antibodies. The middle blots are P0. The bottom blots are β-actin. **a;** ipsilateral side, **b;** contralateral side. The graph presents the optical density ratios of βIII-tubulin/β-actin (open circle) and P0/β-actin (closed square). Data are presented as means ± SEM. **p<0.01, *p<0.05 compared with the intact group.

Toluidine blue staining using transverse sections demonstrated two peaks in the intact myelinated fiber diameter histograms (Fig. 1A–i). The first myelinated fiber diameter peak was between 4 and 7 µm while the second peak occurred in the range of 10–12 µm (Fig. 1A–i). At one week after ligation, the myelinated fiber density was significantly reduced (Fig. 1A–b, e, and h, P<0.01), and dramatic morphological changes including myelin vacuolation and demyelination were clearly observed at both sites (proximal and distal) of the injured nerve (Fig. 1A–b and e). At three weeks after ligation, numerous small regenerated myelinated fibers, but no large myelinated fibers, were observed at the proximal site (Fig. 1 A–c). Both the histogram and the cumulative percentage plot were significantly shifted to the left as compared with those of the intact nerve (Fig. 1 A–j and k, P<0.01). The re-myelinated fiber density was significantly higher than that in the intact nerve (Fig. 1A–h, P<0.01). The vacuolated and degenerating myelin remained at the distal site (Fig. 1A–f). At seven weeks after ligation, larger myelinated fibers were observed at the proximal site (Fig. 1A–d), and the myelinated fiber density was lower than at three weeks after ligation (Fig. 1A–h, P<0.05). Both the histogram and the cumulative percentage plot were significantly shifted to the right as compared with those at three weeks after ligation (Fig. 1A–j and k). At the distal site, numerous small regenerated myelinated fibers were observed (Fig. 1A–g). These results demonstrated that Wallerian degeneration peaked at about one week after ligation, and that regeneration and re-myelination had begun within three weeks after ligation. To confirm these histological results, we performed an immunoblot analysis using anti-βIII-tubulin and anti-myelin protein zero (P0) antibodies. PSL markedly decreased βIII-tubulin and P0 protein expressions on the side ipsilateral to the injured sciatic nerve. The lowest expression was seen at one week after ligation followed by gradual restoration (Fig. 1B).

### Effects of GK on Iba-1 Positive Cell Accumulation in the Injured Nerve

**Figure 2:**
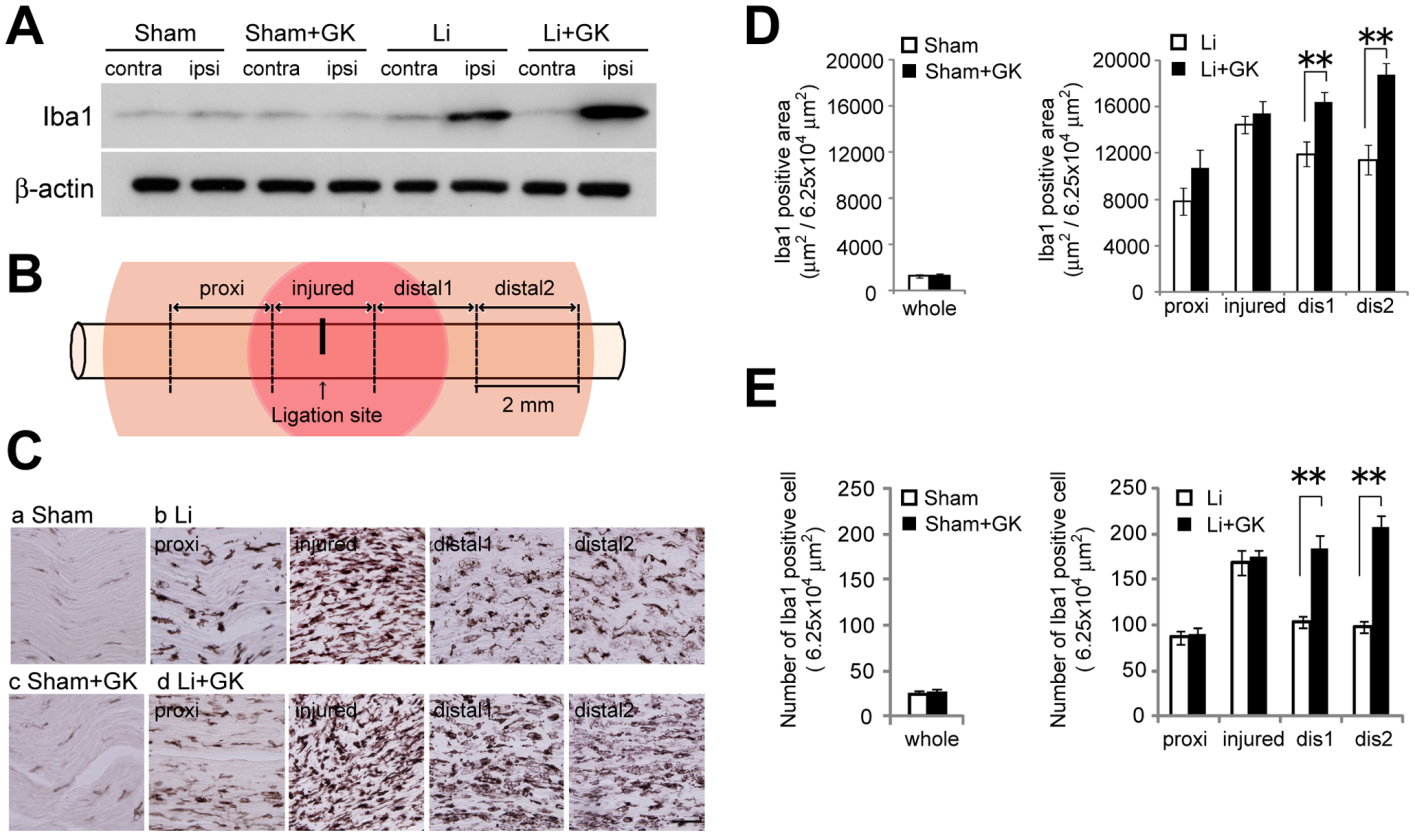
Further accumulation of Iba-1 positive macrophages in the ipsilateral sciatic nerves of PSL rats after GK-irradiation. **A,** Effects of GK irradiation on Iba1 protein expression in the sciatic nerve. The top blots are examples of immunoreactive bands against anti-Iba-1 antibodies. The bottom blots are β-actin. **B,** Schematic figures of a ligation site and the areas analyzed. Histological photographs of injured nerves taken in four areas; proximal site, injured site, distal1 site and distal2 site. Red area; predicted 80% isodose irradiated area. Orange area; predicted 30% isodose irradiated area. **C,** Representative photographs of Iba-1 immunoreactivity on the ipsilateral sciatic nerve after GK-irradiation. **a;** sham-operated rats. **b;** ligation rats. **c;** GK-irradiated sham-operated rats. **d;** GK-irradiated ligation rats. Scale bar; 50 µm. **D,** The size of the Iba-1 positive area in each zone was measured using ImageJ. Data are presented as means ± SEM. **p<0.01 compared with Ligation group. **E,** The number of Iba-1 positive cells in each zone was determined using a cell counter with ImageJ. Data are presented as means ± SEM. **p<0.01 compared with Ligation group.

We performed immunoblotting to elucidate the effects GK (maximum dose of 90 Gy) irradiation on Iba-1 protein expression. Iba-1 protein levels were increased three weeks after ligation and Iba-1 protein in sciatic nerves was further increased by GK irradiation (169.4±11.6% of non-irradiated PSL group, p<0.01). GK treatment had virtually no influence on Iba-1 protein levels in sham-operated rats (97.7±11.5% of non-irradiated sham-operated group, p = 0.98) (Fig. 2A). Further immunohistochemical analysis demonstrated PSL to induce migration of Iba-1 positive cells at the ligation site for three weeks after the procedure. Iba-1 immunoreactive cells had accumulated in the injured sciatic nerve three weeks after ligation (Fig. 2C–a and b). GK irradiation of the injured sciatic nerve induced significant increases in the Iba-1 immunoreactive area and cells distal to the ligation site (Fig. 2C–b, d, 2D, and 2E). In sham-operated rats, Iba-1 positive cells were uniformly scattered along the sciatic nerve trunk and were unaffected by GK irradiation (Fig. 2C–a, c, 2D, and 2E).

### Effects of GK Irradiation on Myelin Debris Clearance

**Figure 3:**
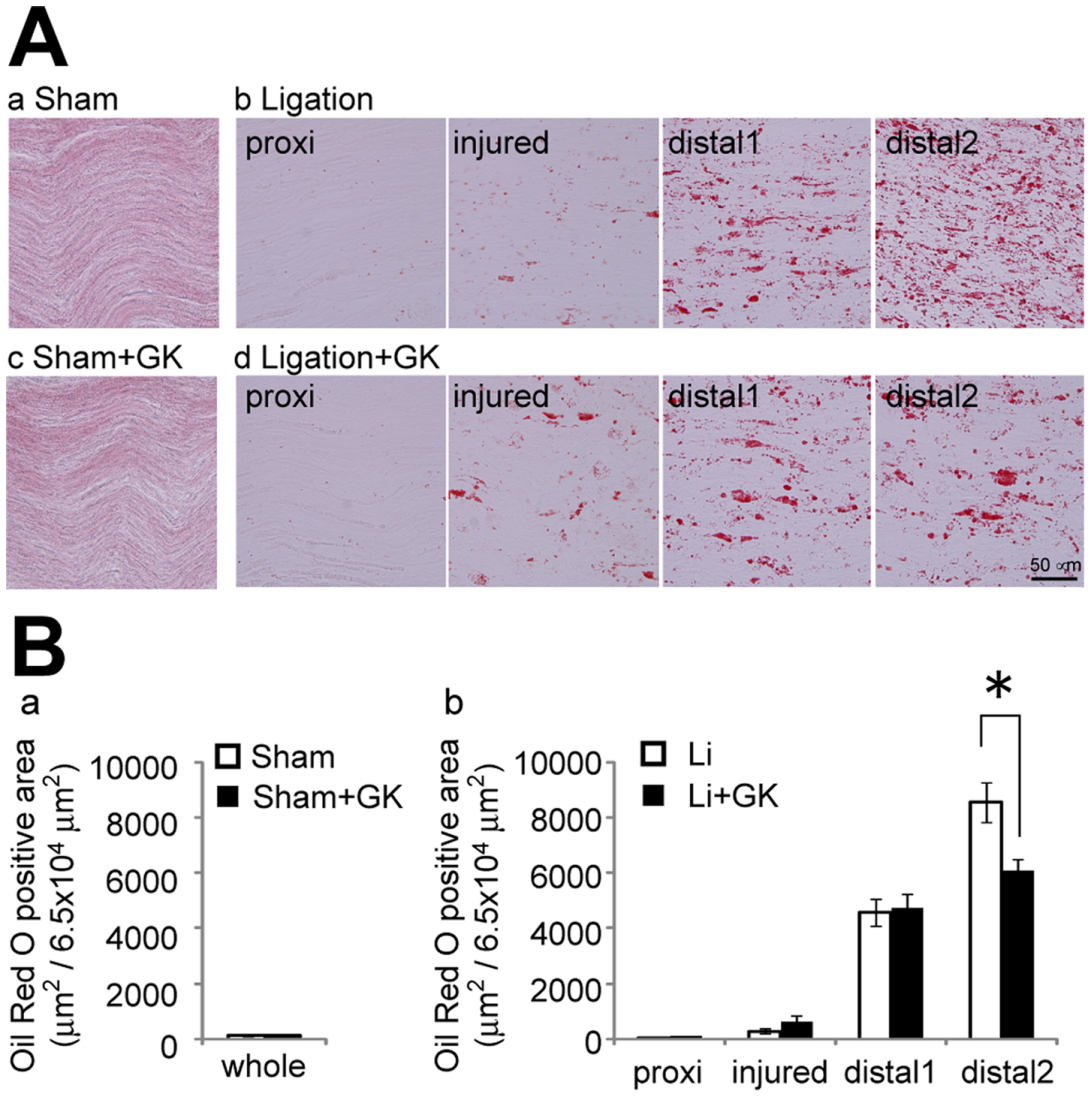
Myelin debris clearance is higher in GK-irradiated than in non-irradiated injured nerves. **A,** Representative photographs of Oil Red O staining of the ipsilateral sciatic nerve after GK irradiation. GK irradiation was performed one week after ligation and sciatic nerve samples were collected three weeks after ligation. **a;** sham-operated rats, **b;** ligation rats, **c;** GK-irradiated sham-operated rats, and **d;** GK-irradiated ligation rats. **B,** The size of the Oil Red O positive area in each zone was measured using ImageJ. Scale bar; 50 µm. Data are presented as means ± SEM. *p<0.05 compared with Ligation group.

To confirm the clearance of myelin debris, longitudinal sections of the sciatic nerve were stained with Oil Red O. As expected, three weeks after ligation, numerous Oil Red O positive follicles were detected in both the injured and the distal areas (Fig. 3A–b). The density of Oil Red O positive follicles gradually increased from the proximal to the distal portion of the nerve (Fig. 3A–b and B–b). In the distal 2 area of injured nerves irradiated with GK, myelin debris was significantly decreased (Fig. 3A–b and d, Fig. 3B–b). Sciatic nerve sections in sham-operated rats were minimally stained by Oil Red O (Fig. 3A–a). There were no significant differences in Oil Red O positive areas between GK-irradiated and sham-operated rats (Fig. 3A–c and Ba).

### Morphological Changes after GK Irradiation of Injured Nerves

**Figure 4:**
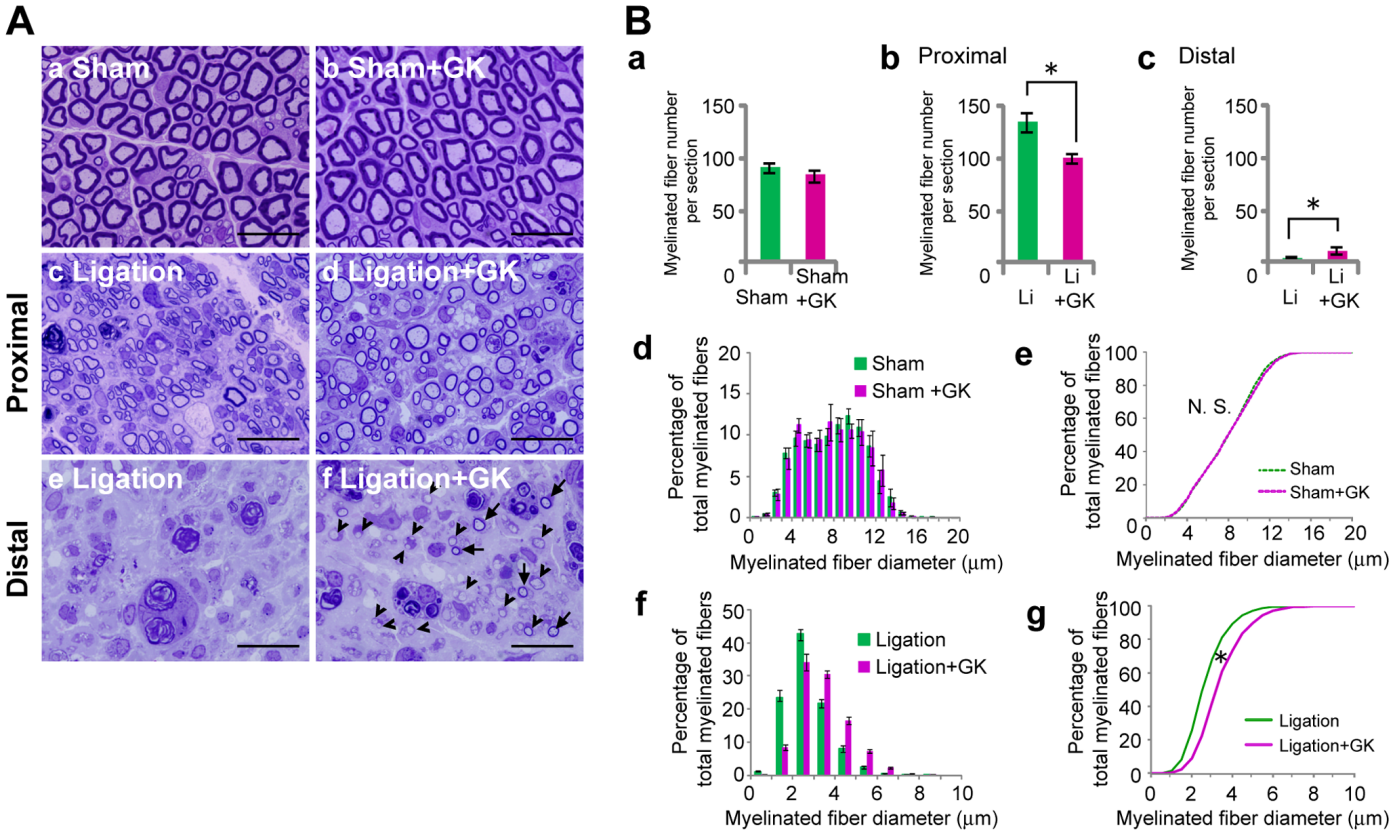
Morphological changes in GK-irradiated injured nerves analyzed by toluidine blue staining. **A,** Representative photographs of toluidine blue stained cross sections of the ipsilateral sciatic nerve after GK-irradiation. Semi-thin cross sections (0.5 µm) of sciatic nerves 1 mm proximal to the injury site (**c** and **d**) or 1 mm distal to the injury site were stained (**e** and **f**) with 0.5% toluidine blue. **a;** sham-operated rats, **b;** GK-irradiated sham-operated rats, **c and e;** ligation rats, and **d and f;** GK-irradiated ligation rats. Scale bars; 20µm for each panel. Arrow; re-myelinated fiber. Arrowhead; immature re-myelinated axon. **B, a-c;** these graphs show the density of myelinated fibers per section from each rat. **a;** sham and sham+GK rats, **b**; proximal sections from ligation and ligation+GK rats, **c;** distal sections from ligation and ligation+GK rats. *p<0.05 compared with non-irradiated nerves from each group. **d and f,** Distribution of myelinated fiber diameters is shown as a histogram with 1µm bin. Data are presented as means ± SEM. **d;** sham and sham+GK rats. **f;** ligation and ligation+GK rats. **e and g,** Cumulative percentage distribution is plotted against the myelinated fiber diameter. **e;** sham (*n* = 3239 fibers) and sham+GK (*n* = 1972 fibers). **g;** ligation (*n* = 6179 fibers) and ligation+GK (*n* = 2386 fibers). *p<0.05 compared with Ligation group.

At the proximal site of the GK-irradiated injured nerve, the population of larger myelinated fibers was increased as compared to that of the non-irradiated PSL group (Fig. 4A–c and d). Both the histogram and the cumulative percentage plot of GK-irradiated injured nerves were significantly shifted to the right (Fig. 4B f and g, p<0.05). The re-myelinated fiber density of GK-irradiated nerves was significantly lower than that of non-irradiated injured nerves (Fig. 4B–b, p<0.05). At the distal site of the GK-irradiated nerve, a few re-myelinated small fibers and numerous immature re-myelinated axons were observed (Fig. 4A e, f, and 4B–c). On the other hand, no morphological changes of myelinated fibers were detected in GK-irradiated sham rats (Fig. 4A a and b, 4B d and e). Similarly, the fiber density was not changed by GK irradiation in sham-operated rats (Fig. 4B–a).

### βIII-tubulin and P0 Protein Expressions after GK Irradiation of Injured Nerves

**Figure 5:**
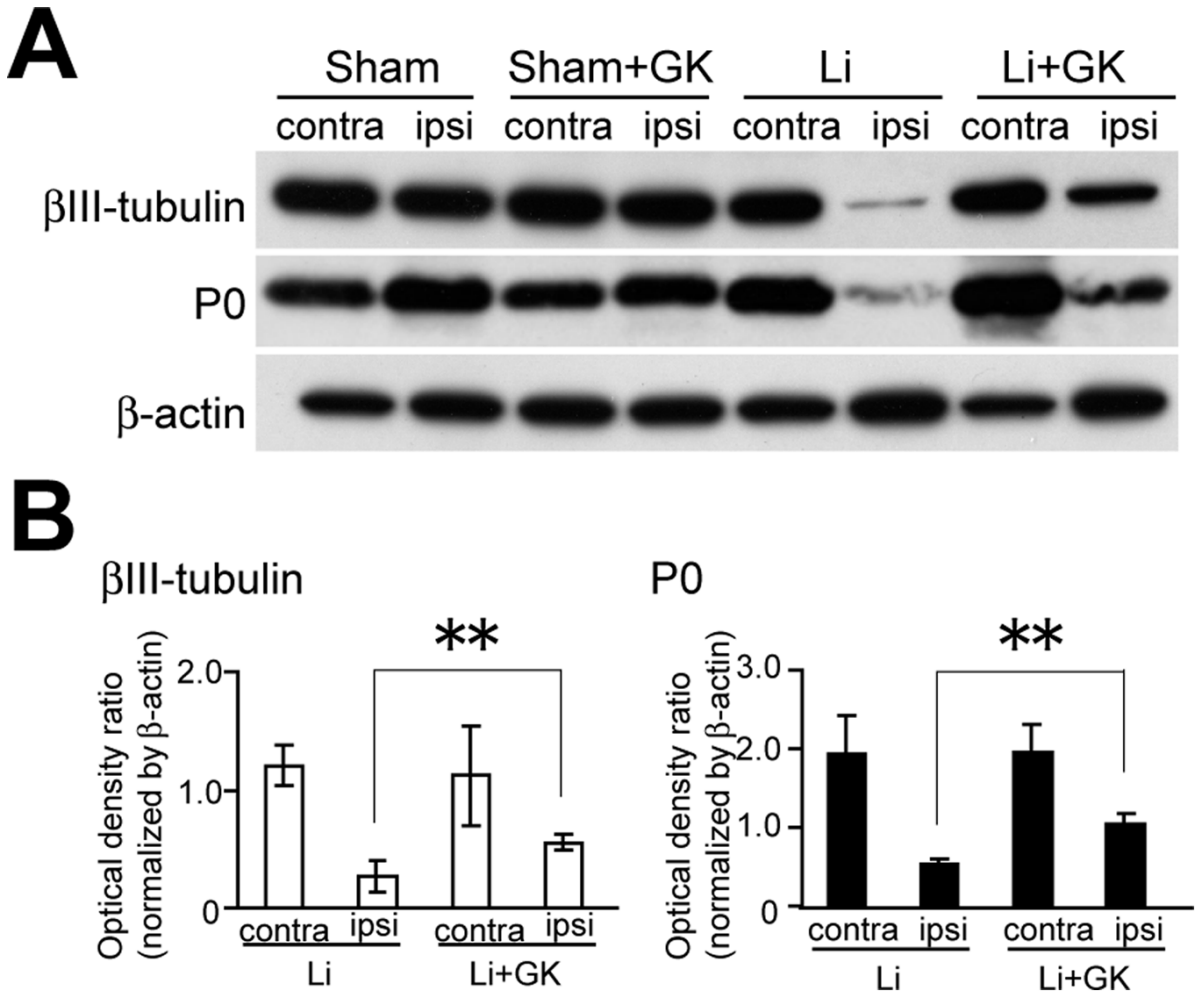
Immunoblot analyses of βIII-tubulin and P0 protein expressions two weeks after GK irradiation of the sciatic nerve. **A,** The top blots are examples of immunoreactive bands against anti-βIII-tubulin antibodies. The middle blots are P0. The bottom blots are β-actin. **B,** One graph presents the optical density ratio of βIII-tubulin/β-actin, the other that of P0/β-actin. Data are presented as means ± SEM. **p<0.01 compared with the non-irradiated group.

βIII-tubulin and P0 protein levels after ligation were higher in GK-irradiated nerves than in ligated but non-irradiated nerves (βIII-tubulin; 270.9±68.7% of the non-irradiated PSL group, p<0.01. P0; 191.2±25.2% of the non-irradiated PSL group, p<0.01). In sham-operated rats, GK treatment had little influence on βIII-tubulin and P0 protein levels (βIII-tubulin; 101.4±14.7% of the non-irradiated sham group, p = 0.95, P0; 94.8±5.9% of the non-irradiated sham group, p = 0.68) (Fig. 5).

### Behavioral Effects of GK Irradiation

**Figure 6:**
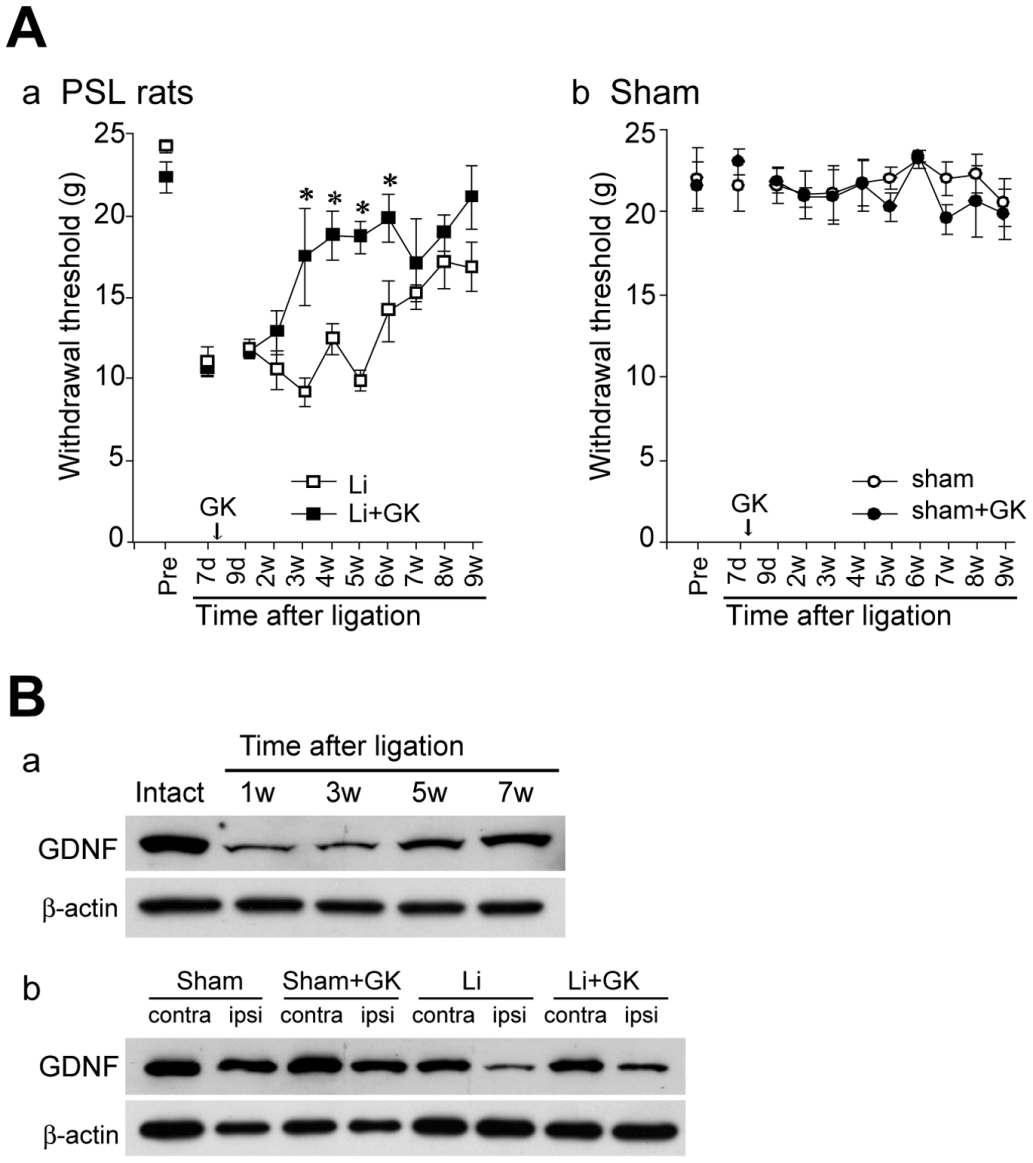
GK irradiation induces anti-allodynic effects in PSL rats and increases GDNF protein expression in injured nerves. **A,** Mechanical withdrawal threshold was determined with a Dynamic Plantar Aesthesiometer. GK irradiation was performed one week post-ligation. GK-irradiated PSL rats showed clear reductions of mechanical allodynia starting at three weeks after ligation (closed square in a). *p<0.05 compared to non-irradiated ligation group. There was no significant difference in withdrawal threshold between the sham (open circle in b) and sham+GK (closed circle in b) groups. Data are presented as means ± SEM. **a;** Ipsilateral side in PSL rats. **b;** Ipsilateral side in sham-operated rats. **B, a;** Time-dependent immunoblot analysis of GDNF protein expressions in the ipsilateral sciatic nerve after ligation. The top blots are examples of immunoreactive bands against anti-GDNF antibodies. The bottom blots are β-actin. **b;** Immunoblot analyses of GDNF expressions two weeks after GK irradiation in the sciatic nerve. The top blots are GDNF. The bottom blots are β-actin.

Tactile allodynia caused by nerve ligation appeared on the ipsilateral side within three days after ligation (data not shown). GK irradiation was performed on PSL rats at one week after ligation. GK-irradiated PSL rats showed more rapid alleviation of allodynia than non-irradiated PSL rats (ligation+GK 3 w; 17.7±3.0 g *versus* ligation 3 w; 9.5±0.9 g, p<0.05) (Fig. 6A–a). In contrast, sham-operated rats did not develop tactile allodynia, and GK had no discernible effects in these rats (Fig. 6A–b).

### GDNF Expression after GK Irradiation of Injured Nerves

We investigated glial cell line-derived neurotrophic factor (GDNF) protein expression in injured nerves. PSL markedly decreased GDNF expressions in the ipsilateral sciatic nerve. The lowest expression was seen at one week after ligation followed by gradual restoration (Fig. 6B–a). In the ipsilateral GK-irradiated nerve, GDNF protein levels were higher than in ligated but non-irradiated nerves (206.0±21.8% of the non-irradiated PSL group, p<0.01). In contrast, GK treatment had little influence on GDNF protein levels in sham-operated rats (91.2±14.6% of the non-irradiated sham group, p = 0.58) (Fig. 6B–b).

## Discussion

In the GK-irradiated injured nerve, the population of larger re-myelinated fibers at the proximal site and the density of small re-myelinated fibers at the distal site were significantly increased as compared with those of non-irradiated injured nerves. These results suggested that GK irradiation facilitated regeneration and re-myelination of injured nerves (Fig. 1A and 5A). Markedly elevated expressions of βIII-tubulin proteins, markers of axonal regeneration [27], [28], [29], and P0 protein expression, a marker of re-myelination [32], suggested that GK irradiation facilitates repair of injured nerves. These results showed GK irradiation to promote regeneration and re-myelination of injured nerves.

Additional macrophage accumulation, as demonstrated by the Iba-1 experiments after GK irradiation, may affect regeneration (Fig. 2 and 3). Macrophages, which reportedly stimulate nerve regeneration in both the peripheral and the central nervous system [33], [34], [35], [36], engulf myelin debris in the early stages of regeneration after nerve injury [37], [38]. An increase in Iba1 positive macrophages and a decrease in myelin debris appeared in the same areas distal to the ligation point at two weeks after GK (Fig. 2 and 3), suggesting that additional macrophages rapidly removed myelin debris and thereby facilitated regeneration.

Neurotrophic factors might participate in the beneficial effects of GK irradiation. Since GDNF increased after GK irradiation in the basal ganglia and possibly exerted a neuroprotective effect [39], we focused on this neurotrophic factor. As expected, GDNF was increased in the injured nerve at two weeks after GK irradiation (Fig. 6B). GDNF, one of the most potent neurotrophic factors influencing the survival and maintenance of neurons [40], [41], has been reported to enhance axonal regeneration and re-myelination following nerve injury [40], [42], [43]. GDNF protein elevation in GK-irradiated injured nerves may promote regeneration and re-myelination. An increase in GDNF expression may also be related to alleviation of allodynia in GK-irradiated PSL rats. The temporal changes in GDNF expression after ligation corresponded to time-dependent behavioral changes indicating alleviation of the allodynia state (Fig. 6). Furthermore, GDNF expression was significantly increased by GK irradiation, when GK-induced alleviation of allodynia was initially detected (Fig. 6). A decrease in the GDNF signal reportedly contributes to the development and/or maintenance of neuropathic pain states [43], [44]. Exogenous GDNF application to the injured area or intrathecal injection induced potent analgesic effects in rat pain models [43], [45]. Therefore, GDNF is the most promising factor among those possibly responsible for the analgesic effects following GK irradiation.

Alleviation of allodynia in GK-irradiated PSL rats may be associated with promoting the regenerative effects induced by GK. We sequentially studied structural and functional changes in the injured peripheral nerves of rats, and confirmed that alleviation of allodynia was accompanied by peripheral nerve morphological improvements (Fig. 1 and 6) as seen in previous experiments [43], [46]. At three weeks after ligation, the analgesic effect appeared only in GK-irradiated PSL rats (Fig. 6), and GK-irradiated injured nerves showed a more mature regenerative morphology than non-irradiated nerves (Fig. 4 and 5).

In our study, the maximum GK-irradiation dose of 90 Gy did not produce detectable nerve damage in sham-operated rats (Fig. 2, 3, 4, 5, 6), while previous studies have found that 70∼100 Gy doses delivered to the trigeminal nerve induced damage including axon degeneration and demyelination [13], [14]. This discrepancy might be explained by the differences in experimental conditions; different species, target nerves and especially observation periods (1 to 7 weeks in our study versus 6 months in prior studies). According to classification of the time courses of radiation-induced effects [47], [48], our experiments pertain to a very early period. In our experiments, GK may have exerted additional effects in the late phase such as at 6 months, because we identified a few Oil Red O positive areas in some portions of the irradiated nerves in the GK-irradiated sham-operated rats. These limitations will be addressed in future studies.

### Conclusion

Our results indicate that GK irradiation (90 Gy) impacts injured sciatic nerves but not sham-operated sciatic nerves during the observation period employed in this study. We demonstrated that GK irradiation promotes regeneration of the injured sciatic nerve, which may correlate with the alleviation of allodynia. GDNF is one of the putative targets via which GK induces these effects. Although further study is needed, GK irradiation may have modulating effects on the actions of glial cells and immune cells, thereby exerting beneficial effects on injured nerves.

## Supporting Information

Figure S1 **GK irradiation of rat sciatic nerve. A,** Photograph of Regis-Valliccioni frame for rat GK irradiation. A rat can undergo both MR imaging and GK in this frame, enabling direct target planning on the rat’s own MR images. **B,** Plan for GK irradiation of the sciatic nerve. The center of the irradiation area was determined with reference to the visible structures on T2-weighted MR images. We used only one isocenter with a 4-mm collimator for GK irradiation, and the irradiated site was on the right sciatic nerve. We used a central maximum dose of 90 Gy. **a, c, e;** Schematic figures of rat fixation in the frame. Corresponding 2D MR image cross sections are indicated by the red square **a;** coronal, **c;** sagittal, and **e;** horizontal. **b, d, f;** Planning on 2D MR images. **b;** coronal, **d;** sagittal, and **f;** horizontal. Green indicates the 80% isodose area, yellow the 50% isodose area, pink the sciatic nerve, and blue the femur.
(TIF)

Figure S2 **Confirmation of the GK-irradiated area.** To determine whether GK irradiation of the rat sciatic nerve was performed correctly, we delivered high-dose irradiation in preliminary experiments. A central maximum irradiation dose of 200 Gy was delivered to the right sciatic nerve (N = 2). Four weeks after 200 Gy irradiation, we determined changes in Iba-1 positive cells, a marker of macrophages and microglia, because glial and immune cells were previously reported to be activated after irradiation [15], [16], [17]. The Iba-1 positive area and cell number were significantly increased in the irradiated sciatic nerve. The maximum increase was observed in ‘area c’, and gradually decreased on the each side of the nerve (Fig S2B-D). The ‘area c’ was a GK irradiation center based on the planning using MR images (Fig S1A). The density changes of Iba-1 positive cells along the nerve (from area ‘a’ to ‘f’) corresponded to the GK irradiation doses. We concluded that GK irradiation was performed with high accuracy in this experiment. **A,** Schematic figure of a sciatic nerve and GK-irradiated area. The predicted 80% isodose area is indicated as a red circle and the predicted 30% isodose area is indicated as yellow circle. Histological photograph of cross sections of sciatic nerves taken in six areas; from ‘a’ to ‘f’. **B,** Representative photograph of Iba-1 immunoreactivity on the right sciatic nerve after delivery of a maximum 200 Gy irradiation dose. Scale bar; 200 µm (upper) and 50 µm (lower). **C,** The size of the Iba-1 positive area in each zone was measured using ImageJ. Data are presented as means ± SEM. **p<0.01, *p<0.05 compared with the non-irradiated nerve. The statistical significance of differences was determined using the repeated measures ANOVA with Dunnett’s multiple comparisons test. **D,** The number of the Iba-1 positive cells in each zone was determined using a cell counter with ImageJ. Data are presented as means ± SEM. **p<0.01, *p<0.05 compared with the non-irradiated nerve. The statistical significance of differences was determined using the repeated measures ANOVA with Dunnett’s multiple comparisons test.
(TIF)

References S1 **References of supporting information.**
(DOCX)

Supplementary Data S1 **Establishment of GK irradiation of rat sciatic nerve.**
(DOCX)
