# Supplementary figures and images for: Gamma Knife Irradiation of Injured Sciatic Nerve Induces Histological and Behavioral Improvement in the Rat Neuropathic Pain Model
Source: PLoS One. 2013 Apr 12;8(4):e61010. doi: 10.1371/journal.pone.0061010 (PMC3625209; doi:10.1371/journal.pone.0061010)

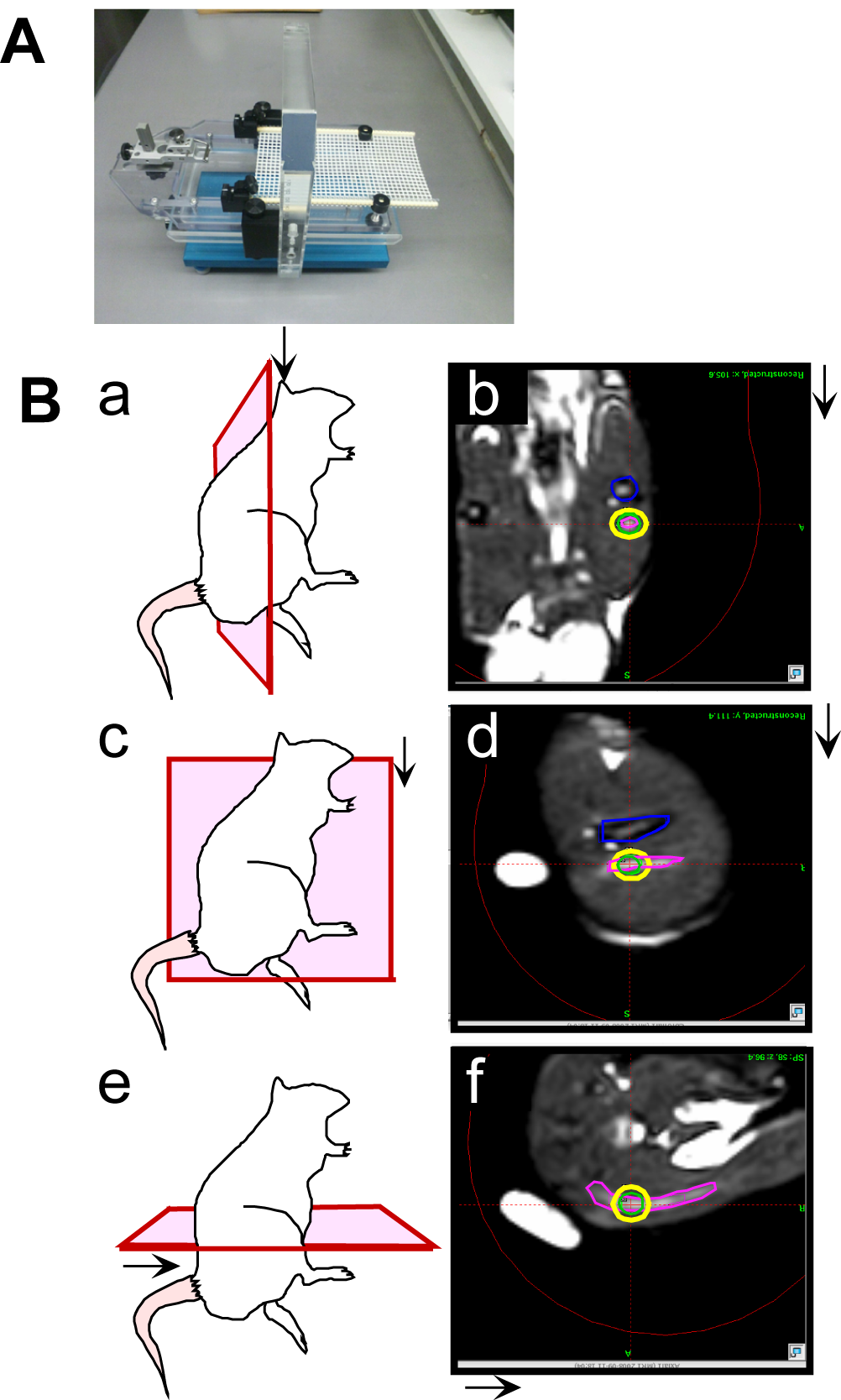

Supplement: Figure S1 — GK irradiation of rat sciatic nerve. A, Photograph of Regis-Valliccioni frame for rat GK irradiation. A rat can undergo both MR imaging and GK in this frame, enabling direct target planning on the rat’s own MR images. B, Plan for GK irradiation of the sciatic nerve. The center of the irradiation area was determined with reference to the visible structures on T2-weighted MR images. We used only one isocenter with a 4-mm collimator for GK irradiation, and the irradiated site was on the right sciatic nerve. We used a central maximum dose of 90 Gy. a, c, e; Schematic figures of rat fixation in the frame. Corresponding 2D MR image cross sections are indicated by the red square a; coronal, c; sagittal, and e; horizontal. b, d, f; Planning on 2D MR images. b; coronal, d; sagittal, and f; horizontal. Green indicates the 80% isodose area, yellow the 50% isodose area, pink the sciatic nerve, and blue the femur. (TIF) [file pone.0061010.s001.tif]

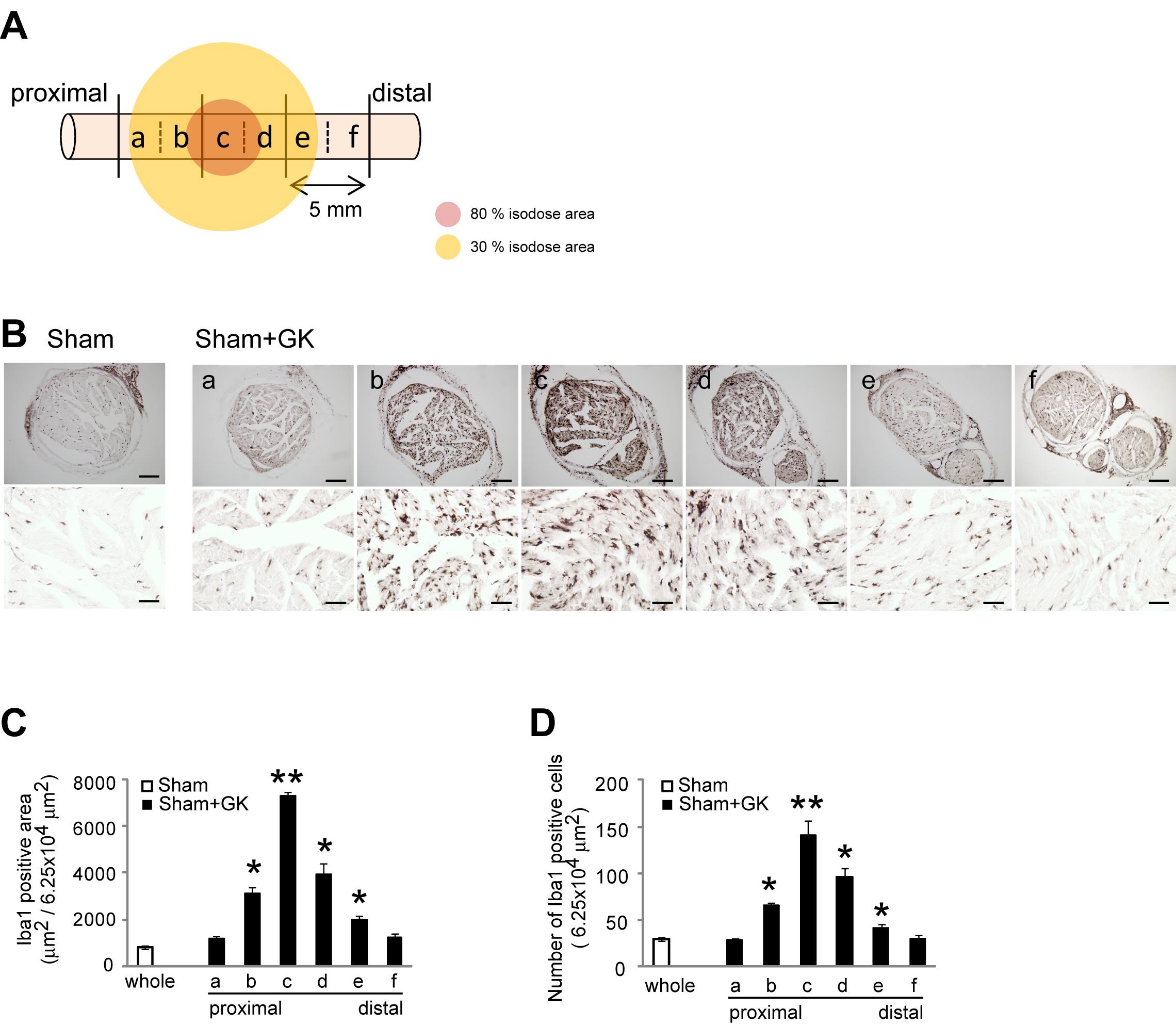

Supplement: Figure S2 — Confirmation of the GK-irradiated area. To determine whether GK irradiation of the rat sciatic nerve was performed correctly, we delivered high-dose irradiation in preliminary experiments. A central maximum irradiation dose of 200 Gy was delivered to the right sciatic nerve (N = 2). Four weeks after 200 Gy irradiation, we determined changes in Iba-1 positive cells, a marker of macrophages and microglia, because glial and immune cells were previously reported to be activated after irradiation [15], [16], [17]. The Iba-1 positive area and cell number were significantly increased in the irradiated sciatic nerve. The maximum increase was observed in ‘area c’, and gradually decreased on the each side of the nerve (Fig S2B-D). The ‘area c’ was a GK irradiation center based on the planning using MR images (Fig S1A). The density changes of Iba-1 positive cells along the nerve (from area ‘a’ to ‘f’) corresponded to the GK irradiation doses. We concluded that GK irradiation was performed with high accuracy in this experiment. A, Schematic figure of a sciatic nerve and GK-irradiated area. The predicted 80% isodose area is indicated as a red circle and the predicted 30% isodose area is indicated as yellow circle. Histological photograph of cross sections of sciatic nerves taken in six areas; from ‘a’ to ‘f’. B, Representative photograph of Iba-1 immunoreactivity on the right sciatic nerve after delivery of a maximum 200 Gy irradiation dose. Scale bar; 200 µm (upper) and 50 µm (lower). C, The size of the Iba-1 positive area in each zone was measured using ImageJ. Data are presented as means ± SEM. **p<0.01, *p<0.05 compared with the non-irradiated nerve. The statistical significance of differences was determined using the repeated measures ANOVA with Dunnett’s multiple comparisons test. D, The number of the Iba-1 positive cells in each zone was determined using a cell counter with ImageJ. Data are presented as means ± SEM. **p<0.01, *p<0.05 compared with the non-irradiated nerve [file pone.0061010.s002.tif]
