# Supplementary material for: Gamma Knife Irradiation of Injured Sciatic Nerve Induces Histological and Behavioral Improvement in the Rat Neuropathic Pain Model
Source: PLoS One. 2013 Apr 12;8(4):e61010. doi: 10.1371/journal.pone.0061010 (PMC3625209; doi:10.1371/journal.pone.0061010)
Supplement: References S1 — References of supporting information. (DOCX) [file pone.0061010.s003.docx]

**References of supplementary data**

1. Reinacher P, Blum C, Gass P, Karger C, Debus J (1999) Quantification of microglial late reaction to stereotactic irradiation of the rat brain using computer-aided image analysis. Exp Neurol 160: 117-123.

2. Kamiryo T, Kassell N, Thai Q, Lopes M, Lee K, et al. (1996) Histological changes in the normal rat brain after gamma irradiation. Acta Neurochir (Wien) 138: 451-459.

3. Yang T, Wu SL, Liang JC, Rao ZR, Ju G (2000) Time-dependent astroglial changes after gamma knife radiosurgery in the rat forebrain. Neurosurgery 47: 407-415; discussion 415-406.
