# Supplementary material for: Gamma Knife Irradiation of Injured Sciatic Nerve Induces Histological and Behavioral Improvement in the Rat Neuropathic Pain Model
Source: PLoS One. 2013 Apr 12;8(4):e61010. doi: 10.1371/journal.pone.0061010 (PMC3625209; doi:10.1371/journal.pone.0061010)
Supplement: Supplementary Data S1 — Establishment of GK irradiation of rat sciatic nerve. (DOCX) [file pone.0061010.s004.docx]

**Supplementary data S1**

**Establishment of GK irradiation of rat sciatic nerve**

We used the Regis-Valliccioni frame for rat sciatic nerve GK irradiation (Fig. S1A). A rat can undergo both MR imaging and GK in this frame, enabling direct target planning on the rat’**s** own MR images. The center of the irradiation area was determined with reference to the visible structures on T2-weighted MR images (Fig S1B). We used only one isocenter with a 4-mm collimator for GK irradiation. To determine whether GK irradiation of the rat sciatic nerve was performed correctly, we delivered high-dose irradiation in preliminary experiments. A central maximum irradiation dose of 200 Gy was delivered to the right sciatic nerve (N=2). Four weeks after 200 Gy irradiation, we determined changes in Iba-1 positive cells, a marker of macrophages and microglia, because glial and immune cells were previously reported to be activated after irradiation [[1](#_ENREF_1),[2](#_ENREF_2),[3](#_ENREF_3)]. The Iba-1 positive area and cell number were significantly increased in the irradiated sciatic nerve. The maximum increase was observed in ‘area c’, and gradually decreased on the each side of the nerve (Fig S2B-D). The ‘area c’ was a GK irradiation center based on the planning using MR images (Fig S1A). The density changes of Iba-1 positive cells along the nerve (from area ‘a’ to ‘f’) corresponded to the GK irradiation doses. We concluded that GK irradiation was performed with high accuracy in this experiment.

**References of supplementary data**

1. Reinacher P, Blum C, Gass P, Karger C, Debus J (1999) Quantification of microglial late reaction to stereotactic irradiation of the rat brain using computer-aided image analysis. Exp Neurol 160: 117-123.

2. Kamiryo T, Kassell N, Thai Q, Lopes M, Lee K, et al. (1996) Histological changes in the normal rat brain after gamma irradiation. Acta Neurochir (Wien) 138: 451-459.

3. Yang T, Wu SL, Liang JC, Rao ZR, Ju G (2000) Time-dependent astroglial changes after gamma knife radiosurgery in the rat forebrain. Neurosurgery 47: 407-415; discussion 415-406.
